# Silencing sensory neuron membrane protein *RferSNMPu1* impairs pheromone detection in the invasive Asian Palm Weevil

**DOI:** 10.1038/s41598-024-67309-x

**Published:** 2024-07-17

**Authors:** Jibin Johny, Mohammad Nihad, Hattan A. Alharbi, Mohammed Ali AlSaleh, Binu Antony

**Affiliations:** 1 Department of Plant Protection, Center for Chemical Ecology and Functional Genomics, College of Food and Agricultural Sciences, King Saud University, 11451 Riyadh, Saudi Arabia; 2 Present Address: Faculty of Forestry and Wood Sciences, Czech University of Life Sciences, Prague, Czechia

**Keywords:** Palm weevil, Olfaction, Sensory neuron membrane protein, RNAi, Electrophysiology, Pest control, Ecology, Evolution, Neuroscience, Zoology

## Abstract

The red palm weevil (RPW), *Rhynchophorus ferrugineus* (Olivier), also known as the Asian palm weevil, is an invasive pest that causes widespread damage to palm trees around the globe. As pheromone communication is crucial for their mass attack and survival on palm trees, the olfactory concept of pest control strategies has been widely explored recently. We aim to understand the molecular basis of olfaction in RPW by studying one of the key olfactory proteins in insect pheromone communication, sensory neuron membrane proteins (SNMPs). SNMPs belong to the CD36 (cluster of differentiation 36) family that perform two distinct olfactory roles in insects, either in pheromone (odorant) transfer to the odorant receptors (SNMP1) or in the pheromone clearing process (SNMP2). In this study, we performed antennal transcriptomic screening and identified six SNMPs, mapping them on the *R. ferrugineus* genome, and confirmed four distinct SNMPs. Both SNMP1 proteins in RPW, viz*., RferSNMPu1* and *RferSNMPu2,* were mapped onto the same scaffold in different loci in the RPW genome. To further understand the function of these proteins, we first classified them using phylogenetic analysis and checked their tissue-specific expression patterns. Further, we measured the relative transcript abundance of SNMPs in laboratory-reared, field-collected adults and pheromone-exposure experiments, ultimately identifying *RferSNMPu1* as a potential candidate for functional analysis. We mapped *RferSNMPu1* expression in the antennae and found that expression patterns were similar in both sexes. We used RNAi-based gene silencing to knockdown *RferSNMPu1* and tested the changes in the RPW responses to aggregation pheromone compounds, 4-methyl-5-nonanol (ferrugineol) and 4-methyl-5-nonanone (ferrugineone), and a kairomone, ethyl acetate using electroantennogram (EAG) recordings. We found a significant reduction in the EAG recordings in the *RferSNMPu1* knockdown strain of adult RPWs, confirming its potential role in pheromone detection. The structural modelling revealed the key domains in the *RferSNMPu1* structure, which could likely be involved in pheromone detection based on the identified ectodomain tunnels. Our studies on *RferSNMPu1* with a putative role in pheromone detection provide valuable insight into understanding the olfaction in *R. ferrugineus* as well as in other Curculionids, as SNMPs are under-explored in terms of its functional role in insect olfaction. Most importantly, *RferSNMPu1* can be used as a potential target for the olfactory communication disruption in the *R. ferrugineus* control strategies.

## Introduction

The Asian Palm Weevil or Red Palm Weevil (RPW), *Rhynchophorus ferrugineus* (Olivier), is an invasive insect pest originating in Southeast Asian countries. In the past few decades, RPW has reached a widespread dispersion to various regions across the globe, encompassing North Africa, the Middle East, the Mediterranean region, and portions of the Caribbean and Central America^1,2^. This infestation has resulted in the devastating loss of numerous palm farms in different countries, making it a severe agricultural problem^3–6^. For example, Gulf and Middle Eastern countries annually face an expenditure of $8 million dedicated to removing infested palm trees. Food and Agriculture Organization (FAO) of the United Nations Food Chain Crisis, Early Warning Bulletin reported that RPW is causing widespread damage to date palm *Phoenix dactylifera* and the Canary Island palm *Phoenix canariensis*, affecting palm production and quality, has a negative impact on the livelihoods of farmers and the environment^7^.

The primary mechanism behind the infestation of RPW is its capacity to locate host palm trees and generate a mass attack typically accomplished and guided through male-produced aggregation pheromone, composed of 4-methyl-5-nonanol (ferrugineol) and 4-methyl-5-nonanone (ferrugineone)^8^. Therefore, detecting this species-specific aggregation pheromone is crucial for RPW survival^9,10^. In general, peripheral olfactory reception involving classical olfactory family proteins, viz*.*, odorant-binding proteins (OBPs), odorant receptors (ORs), and odorant receptor coreceptor (Orco), plays a pivotal role in odorant detection^11^. Recent studies on RPW unraveled the molecular basis of pheromone detection in RPW^9,10,12^. However, these studies are limited to the functional characterization of OBPs, OR, and Orco. At the molecular level, apart from OBPs, OR, and Orco, other olfactory proteins like sensory neuron membrane proteins (SNMPs) and ionotropic receptors (IRs) are actively involved in the chemoreception mechanism^11,13–16^. The SNMPs (pfam01130) are a distinct group of insect olfactory proteins that belong to the CD36 (cluster of differentiation 36) family of proteins in vertebrates^17–20^. It is located in the cilia and dendrites of olfactory receptor neurons (ORNs); SNMPs can bind with proteins and fatty acids^17,20–25^. Recent studies have demonstrated that the proximity between SNMP and OR is critical for the increased sensitivity of neurons responsive to pheromones^25^. Two SNMP families, viz*.*, SNMP1 and SNMP2, have been identified in insects^11^, and the SNMP1 is primarily antennae-specific, expressed in the ORNs^26,27^ whereas SNMP2s are mainly expressed in the non-neuronal support cells of the ORNs^28,29^. The distinctive gene expression patterns suggest that SNMP1 and SNMP2 may have distinct roles in chemoreception^18,26,28,30^. Studies unraveled its expression in the dendritic membrane of neurons^27^ and predominately found co-expression with ORs^20,26^. The SNMP1-OR protein complex is hypothesized to serve as a ‘docking site’ for OBPs carrying pheromones, thereby assisting either in unloading pheromones from the OBPs or transferring the odorant molecules to ORs^31,32^.

To improve our comprehension of the SNMPs associated with palm weevil pheromone detection, we first annotated the *R. ferrugineus* SNMPs (hereafter RferSNMPs) in the recently generated antennal transcriptome data^34^. We generated RferSNMP expression profiles in the male and female antennae, both laboratory-reared and field-collected adults with marked gene positions on the *R. ferrugineus* genome. We performed the phylogenetic analysis to determine the functionally divergent groups of SNMPs followed by tissue-specific and relative expression analyses. Further, we conducted pheromone pre-exposure experiments^10^ on laboratory-reared RPW adults to test whether this exposure would affect the constitutive expression levels of the antennae-enriched RferSNMPs. Taken together, we found a highly expressed, antenna-enriched *RferSNMPu1* as a potential candidate for functional characterization. The structural predictions were used to study the functional domains and its possible interactions with other olfactory proteins. Further, in vivo functional characterization of *RferSNMPu1* was conducted using RNA interference (RNAi) based gene silencing, followed by electrophysiology experiments. Our results highlight the SNMP1’s functional role in pheromone detection in *R. ferrugineus* and offer a novel target for developing next-generation pest control strategies for the most destructive pest of palm trees that inflicts significant economic losses worldwide.

## Materials and methods

### Insect collection and colony maintenance

The RPW original culture was established in 2009; since then, we maintained RPW culture on sugarcane stems, as previously described^10,33^ denoted as lab-reared RPWs. For the current study, the RPW adults were collected directly from the date palm field in the Al-Kharj region (24.1500°N, 47.3000°E) of Saudi Arabia. They were maintained in our laboratory as previously described^10^ and denoted as field-collected RPWs.

### RferSNMP annotation, expression mapping, gene structure analysis

The previously reported six SNMP sequences from *R. ferrugineus*^33^ were screened in the newly generated RPW male and female antennal transcriptomes^34^. The antennal transcriptome data (field-collected and laboratory-reared RPWs) were obtained from the NCBI under BioProject PRJNA275430 and SRA accession numbers: SRR22098129; SRR22098128, SRR22098127, and SRR22098126^34^ were screened and annotated for SNMP candidate genes. We followed the method^34^ and conducted the antennal transcriptome assembly and annotation in the Qiagen CLC Genomics Server (CLC) (*v* 21.0.1). In the raw data, the Illumina adaptors by an automatic read-through adapter trimming option are implemented in the ‘Trim Reads’ tool of the CLC Genomics Server. Filtered paired-end reads were QC validated through a ‘QC for Sequencing Reads Tool' of CLC. We constructed a reference de novo transcriptome assembly with the ‘De Novo Assembly Tool’ of CLC, and after that, contigs were functionally annotated using the BLAST2GO command line tool (*v*1.5). The cleaned RNA-seq reads mapped to the *R. ferrugineus* genome (GenBank assembly accession: GCA_014462685.1) using the ‘RNA-seq analysis procedure’ implemented in CLC. Further, the SNMP sequences were correctly annotated and mapped to the *R. ferrugineus* genome (GenBank accession numbers GCA_014462685.1 and GCA_014490705.1) using a BLASTN search against the *R. ferrugineus* genome created on Geneious v7.1.9 (Biomatters) and correctly annotated. To verify the identified candidate SNMPs and check the open reading frames (ORFs), we used the NCBI BLASTx homology search and ORF Finder (https://www.ncbi.nlm.nih.gov/orffinder/). For convenience, we added a prefix, Rfer (*R. ferrugineus*), for SNMP transcripts, followed by a contig/unigene letter and identification number. Gene expression levels were quantified and reported as reads per kilobase of transcript per million mapped reads (RPKM) and transcripts per kilobase of exon model per million mapped reads (TPM). The expression levels of the transcripts were expressed as normalized TPM values of RferSNMPs using male *vs.* female and laboratory vs. field *R. ferrugineus* transcriptomes using pheatmap (*v*1.0.12) R package. We visualized the expression level and differential expression analysis (male *vs.* female and lab *vs.* field) of RferSNMPs conducted in the CLC Genomics Server. Our objective was to differentiate the total exon and intron read; hence, the expression mapping will accurately predict highly expressed or over-expressed SNMPs in males and females under different conditions (lab-reared vs. field-collected). Differential expression of SNMPs was calculated using the criteria |log2 fold change |≥ 1^35^, False Discovery Rate (FDR) ≤ 0.001^36^, and Bonferroni post-hoc analysis. Normalized TPM values of SNMPs were calculated using male vs. female and laboratory vs. field *R. ferrugineus* antennal transcriptomes.

We mapped the exon-intron positions of RferSNMPs in the genome (GenBank: GCA_014462685) at the scaffold region in a different locus. The mapped regions were extracted and manually aligned using the MAFFT program *v*7^38^, which was used for gene structure illustrations. We used the SIAS tool (http://imed.med.ucm.es/Tools/sias.html) to calculate amino acid similarity and identity. To predict the theoretical pI (isoelectric point) and MW (molecular weight), Compute pI/Mw (http://web.expasy.org/compute_pi/) was used. *RferSNMPu1* transmembrane topology prediction was performed using DeepTMHMM^37^.

### RferSNMP phylogeny and tissue-specific expression analysis

The SNMP protein sequences from closely related species were included in the phylogenetic analysis. For the classification, SNMP sequences representing two separate SNMP classes, SNMP1 and SNMP2^38^, and representatives of newly reported SNMP sub-classes^39^ were used to reconstruct phylogenetic trees. Additionally, a non-SNMP CD36 protein family member, Croquemort (*Crq*) protein, from *Drosophila melanogaster,* was used as an outgroup^40^. Multiple sequence alignment was performed using MAFFT *v*7^41^ under the E-INS-i iterative refinement method. The maximum likelihood phylogenetic tree was reconstructed using LG + R as the best-fit amino acid substitution model under the Bayesian information criterion with 1000 bootstrap replications using IQ-TREE *v*2.2.0^42^.

Antennae, snout, abdomen, legs, thorax, and wings were excised from 20 day-old adult insects for tissue-specificity and qRT-PCR studies. PureLink RNA Mini Kit (Ambion, USA) was used for total RNA extraction from 30 mg of tissue for each sample, and first-strand cDNA was synthesized using SuperScript IV Reverse Transcriptase (Invitrogen, Carlsbad, CA, USA) following the manufacturer’s protocol. NanoDrop spectrophotometer (Thermo, Delaware, USA) was used to examine the quality and quantity of the RNA and cDNA. Primer3 software^43^ was used for primer designing with the following parameters: Tm, 56–60 ℃; GC content, 40–50%; and with product size, 190–200 bp (Table S1). Touchdown polymerase chain reaction (PCR) [95 ℃ for 5 min, 35 cycles of 95 ℃ for 1 min, 60 ℃ (touchdown to 54 ℃) for 30 s and 72 ℃ for 30 s; and one cycle at 72 ℃ for 10 min] was carried out using GoTaq Green PCR Master Mix (Promega, USA), and the PCR products were evaluated by 2.5% agarose gel electrophoresis alongside a 100-bp DNA ladder (Solis BioDyne, Tartu, Estonia) as a marker and visualized using ethidium bromide (Promega, USA) staining.

### Pheromone pre-exposure experiments and relative quantification of RferSNMPs expression

Pheromone pre-exposure experiments and SNMP overexpression analysis on the adult RPWs were conducted using lab-reared overnight starved RPW adults, as the following method mentioned previously^10^. We used two groups of RPW samples, field-collected and laboratory-reared, to achieve higher reliability when comparing SNMP expressions. Specifically, we compared field samples with pheromone pre-exposed groups, as it is assumed that the SNMP involved in pheromone detection would have a higher expression in natural (field) populations than in lab-raised colonies. Briefly, overnight starved 5-day-old adult male and female weevils in separate stimulus containers were exposed to a commercial aggregation pheromone containing 4-methyl-5-nonanol (ferrugineol) and 4-methyl-5-nonanone (ferrugineone) at the approximate ratio of 9:1 (ChemTica Int., Costa Rica). We used a customized olfactometer setup (Volatile Collection System Co, Gainesville, FL) consisting of a Y-tube (main-tube length: 47 cm; arm length: 68 cm; diameter: 5 cm; with 40 cm-long/2 cm-diameter plastic tubes in each arm connected to the source of the stimulus), an air-delivery system (humidified air and carbon filter), and a stimulus container (diameter: 8 cm, length: 10 cm). Ten male and female adults were isolated in a separate stimulus container for the three replicates. They were individually exposed to pheromone stimuli in the container for 4 h during the photophase, at 27 ± 4 ℃ and 60 ± 10% RH, until antennal dissection and RNA extraction. Stimulus consisted of charcoal-filtered, humidified air (0.2 L.min^−1^) passing through a Pasteur pipette containing a piece of filter paper loaded with 1 µg of the synthetic pheromone (ChemTica Int., Costa Rica). Total RNA was extracted, cDNAs were prepared from RNA (~ 1 µg), and qPCR was performed by using ABI-7500 FAST Thermal Cycler. For non-pheromone-induced experimental groups, cDNAs were prepared from RNA (~ 1 µg) extracted from the antennae of 20-day-old insects. The RT-qPCR experiments were performed according to the Minimum Information Required for Publication of Quantitative Real-Time PCR Experiments (MIQE) Guidelines. According to the manufacturer’s instructions, qRT-PCR was carried out using SYBR Green PCR Master Mix (Life Technologies, USA) with three biological and three technical replicates^9^. The oligonucleotide primers were used as in the tissue-specific studies, and gene expression was normalized using *Tubulin* and *β-actin*^10^. The relative RferSNMP expression levels were measured by the 2^−ΔΔCT^ method^44^. The following thermal program was used to perform the PCR amplification: holding stage at 50 °C; 95 ℃ for 2 or 5 min; 40 cycles of 95 ℃ for 15 s; and 60 ℃ for 32 s; and a continuous melting curve stage of 95 ℃ for 15 s, 60 ℃ for 1 min, 95 ℃ for 30 s, and 60 ℃ for 15 s. The qRT-PCR products were then examined by 3% agarose gel electrophoresis and visualized by ethidium bromide staining.

### RferSNMPu1 cloning, structural and in vivo functional analysis

To obtain the full-length (open reading frame, ORF) sequences of candidate SNMPs, the SMARTer rapid amplification of cDNA ends technique (SMARTer RACE Kit, Clontech, CA, USA) was used by amplifying both cDNA ends (5′ and 3′ ends). The 5′ and 3′ RACE cDNAs were prepared from the total RNA of adult *R. ferrugineus* antennae, as described^12^. Gene-specific primers (GSPs) for 5′- and 3′-RACE were designed based on partial *RferSNMPu1* nucleotide sequences. The amplification reactions were carried out as follows: 95 ℃ for 5 min; 30 cycles of 95 ℃ for 1 min, 65 ℃ (touchdown to 60 ℃) for 30 s and 72 ℃ for 2 min; and one cycle at 72 ℃ for 10 min. The amplified PCR products were purified using Wizard SV Gel Purification Kit (Promega, USA) and cloned into the pGEM-T vector (Promega, USA), followed by transformation into JM109 competent cells (Promega, USA). The plasmids were isolated from bacteria, sequenced in both directions (ABI 3500, Life Technologies, MD, USA), aligned, and annotated using a BLASTx homology search.

For structural analysis of *RferSNMPu1* and studying its interactions with other RPW olfactory proteins involved in pheromone communication, we predicted multimeric models of *RferSNMPu1, RferOR1,* and *RferOrco* using Alphafold2^45,46^. The best-ranked model based on Mean pLDDT values (predicted local-distance difference test) for monomeric, and the predicted template modelling (pTM) score^46^ for multimeric was used for visualization using PyMol (https://pymol.org/2/) and UCSF ChimeraX v1.6.1^47^. However, the long C-terminal cytosolic tail, with a low pLDDT score, was excluded from visualization. The predicted *RferSNMPu1* structure was further analyzed for possible tunnels within the ectodomain using Caver Analyst 2.0^48,49^ with 0.09 as the minimum probe radius and the desired radius as 5. The catalytic sites were ranked based on predicted draggability, and tunnels were ranked by their bottleneck radius. All top ranked tunnels within the ectodomain were used for visualization.

We followed the previously described method^9,10,12^ and conducted in vivo functional analysis of *RferSNMPu1* using RNA Interference (RNAi). To synthesize double-stranded RNA (dsRNA), we used plasmids containing the full-length *RferSNMPu1* ORF as template DNA. Plasmids were linearized using ORF-specific primers and were rechecked by direct sequencing (ABI 3500, Life Technologies, USA). MEGAscript RNAi Kit (Life Technologies, USA) was used for dsRNA synthesis, following the manufacturer’s instructions and a previously described method^9,10,50^. The integrity and efficiency of dsRNA duplex formation were evaluated by 1% agarose gel electrophoresis. We then selected approximately 10 day-old *R. ferrugineus* pupae for RNAi experiments, and 40 ng/μL dsRNA (in 20 μL) was injected at a depth of 0.5 cm into the first dorsal segment of the abdomen, close to the thorax, using a 0.5-mL BD Micro-Fine™ PLUS syringe (Becton, Dickinson Co., NJ, USA). dsRNA-injected RPW pupae were maintained as previously described^9,12^. As two separate groups, RPW pupae were injected with dsRNA control (Integrated DNA Technologies, Leuven, Belgium), and not-injected (hereafter referred to as ‘Lab control’) were maintained as controls. The adults emerging at 14–21 days were further subjected to electrophysiological recording using an electroantennogram (EAG), followed by gene silencing quantification (qRT-PCR) as described earlier^9,10^.

### Gene silencing validation by RT-qPCR and electroantennography (EAG)

For RT-qPCR verification transcript knockdown, RNA was extracted (PureLink RNA Mini Kit, Thermo Fisher), and cDNAs were synthesized (SuperScript IV Reverse Transcriptase, Thermo Fisher) from the antennae of individual insects used in the experimental (RferSNMPu1 dsRNA injected) and control (dsRNA control and Lab control) groups following the manufacturer’s protocols. Reactions were carried out using SYBR Green PCR Master Mix (Life Technologies, USA) according to the manufacturer’s instructions in the Applied Biosystems 7500 Fast qPCR System (Thermo Fisher), with three biological and three technical replicates. *Tubulin* and *β-actin*^9,10^ were used to normalize gene expression. The relative expression levels of SNMPs in the silenced vs. control groups were measured by the 2^−ΔΔCT^ method^44^.

The adult insects were subjected to EAG recordings to validate the effect of gene silencing on RPW’s antennal response towards pheromones. A total of six adult RPWs were tested per group (RferSNMPu1 dsRNA injected, dsRNA control, and Lab control) at the age of 21 days. After immobilization using CO_2_ for 1–2 min, the antennae of each insect were excised from the base. Each antenna was then attached to the electrode holders of an EAG system (Syntech, Hilversum, Netherlands) using SPECTRA 360 electrode gel (Parker Lab, Inc. Fairfield, NJ, USA) and subjected to a constant flow of humidified air. Each insect from the experimental groups was exposed to three different stimuli, (4*RS*, 5*RS*)-4-methylnonan-5-ol, (Phe1) (> 92% purity, ChemTica Int., Costa Rica), 4(*RS*)-methylnonan-5-one (Phe2) (> 92% purity, ChemTica Int., Costa Rica), and ethyl acetate (Sigma Aldrich, USA), at concentrations of 0.02 mg/mL (diluted in *n*-hexane). We used a glass Pasteur pipette with a filter paper strip inside (with 4 μL of the stimulus compound) to deliver the stimulus via an air-stimulus controller (Model CS-55 Ver.2.7, Syntech, Hilversum, Netherlands) fitted with a charcoal filter. Odor stimulation puffs were applied twice at 0.1-s intervals and with 20–30 s intervals between each odor compound. The antennal response to each stimulus was recorded using a Syntech Acquisition IDAC-2 controller connected to a computer and processed using GC-EAD 2012 *v*1.2.4 (Syntech, Kirchzarten, Germany).

### Statistical analysis

For the qRT-PCR experiments, mean fold change 2^−ΔΔCT^ values were calculated using the formula^51^ in MS Excel (Microsoft Corporation, USA). Ct values were collected from three experimental groups: dsRNA RferSNMPu1-injected, dsRNA control, and Lab-control groups in triplicate sets as biological and technical replicates. One-way analysis of variance (ANOVA) was used to assess the significant differences among the experimental groups for qRT-PCR and EAG, followed by multiple-comparison testing with the least significant difference (LSD) test (*p* < 0.05) using SPSS program *v*24 (IBM SPSS Statistics, NY, USA).

## Results

### RferSNMP annotation, expression mapping, gene structure analysis

Previously, six RferSNMPs (*RferSNMPu1, RferSNMPu2, RferSNMPc18799, RferSNMPc17112, RferSNMPc21604,* and *RferSNMPc928*) were reported from *R. ferrugineus* antennae^33^. With newly generated antennal transcriptomes SNMP screening and mapping onto the *R. ferrugineus* genome, we confirmed four SNMPs in *R. ferrugineus *viz*.*, *RferSNMPu1, RferSNMPu2, RferSNMPc18799,* and *RferSNMPc17112,* after excluding *RferSNMPc21604* and *RferSNMPc928,* from the previous annotation^33^ as identical sequences. *RferSNMPc21604* was identical to *RferSNMPc18799* (with 99.41% sequence identity), and *RferSNMPc928* was identical to *RferSNMPu2* (with 99.56% sequence identity).

The ORFs obtained were 1527 bp, 1656 bp, 3717, and 1020 bp, respectively, in length for the *RferSNMPu1, RferSNMPu2, RferSNMPc18799*, and *RferSNMPc17112* cDNA transcripts, which correspond to predicted proteins with 544, 551, 1238 and 339 amino acids in length; and 56.12, 62.67, 140.30 and 39.11 kilodaltons (kDa) in predicted molecular weight. We retrieved the protein domain family^52^ of SNMPs, a member of the superfamily cl10574 and CD36 family (pfam01130), thought to be a novel class of scavenger receptors and a possible role in signal transduction.

**Figure 1:**
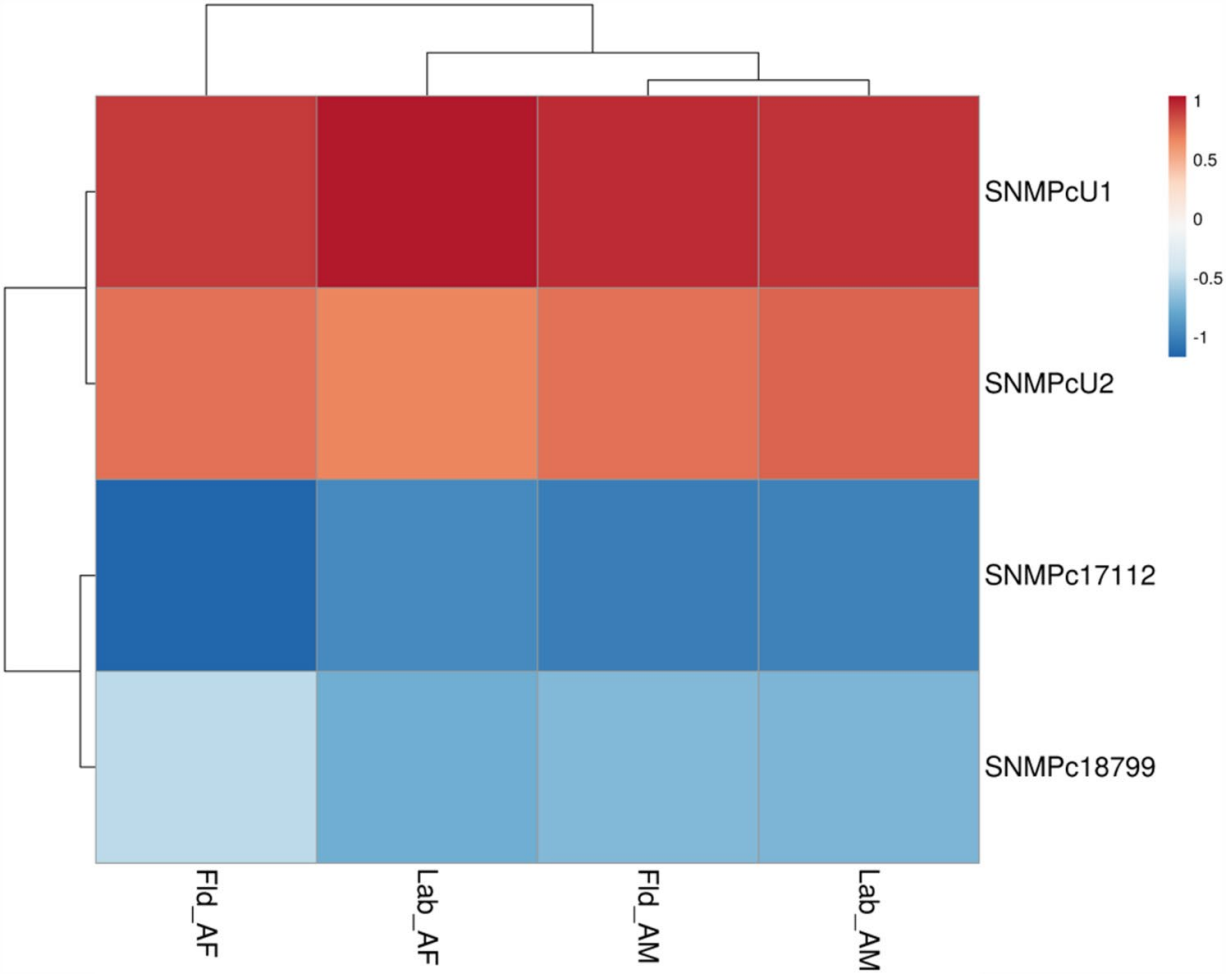
Heatmap showing changes in RferSNMP expression levels in *R. ferrugineus* antennae for lab-reared and field-collected male and female adults. The heatmap colors represent transcript abundance in transcript per million (TPM) from highest (red) to lowest (blue) expression levels. The data represented as log-transformed TPM values tabulated and converted to heatmaps using pheatmap (*v*1.0.12) R package. Fld, insects collected from a date palm field in Saudi Arabia—AM, antenna male, and AF, antenna female.

SNMPs were then classified based on their expression [transcript per million (TPM) values] in the male and female transcriptomes of laboratory-reared RPWs versus field-collected RPWs (Supplementary Tables S2, S3). Both *RferSNMPu1* and *RferSNMPu2* were expressed higher in the antennae of adult males and females in lab and field conditions (Supplementary Tables S2, S3). The log-transformed TPM values clearly defined *RferSNMPu1* as the highly expressing RferSNMP in both lab-reared and field-collected transcriptomes separately generated from RPW males and females (Fig. 1). We mapped the SNMPs’ exon-intron positions in the RPW genome (GenBank: GCA_014462685). Based on the total exon reads of each SNMP in both male and female antennal transcriptome (lab-reared and field-collected), we noticed the highest expression of *RferSNMPu1*, followed by *RferSNMPu2* (Supplementary Tables S4, S5). The expression values of *RferSNMPc18799* and *RferSNMPc17112* were significantly lower than those of *RferSNMPu1* and *RferSNMPu2* in both male and female adult antennae (Supplementary Tables S4, S5). Based on the expression values and fold changes in expression, *RferSNMPu1* expression patterns between male and female samples were not significantly different (Table S2) (Fold change: 1.407762915; *p-value:* 0.43086413; FDR *p*-value: 0.999990455). *RferSNMPu2* shows higher expression in female adults (Fold change: −1.164660322; *p-value:* 0.738887996; FDR *p*-value: 0.999990455) than in males (Supplementary Table S2).

**Figure 2:**
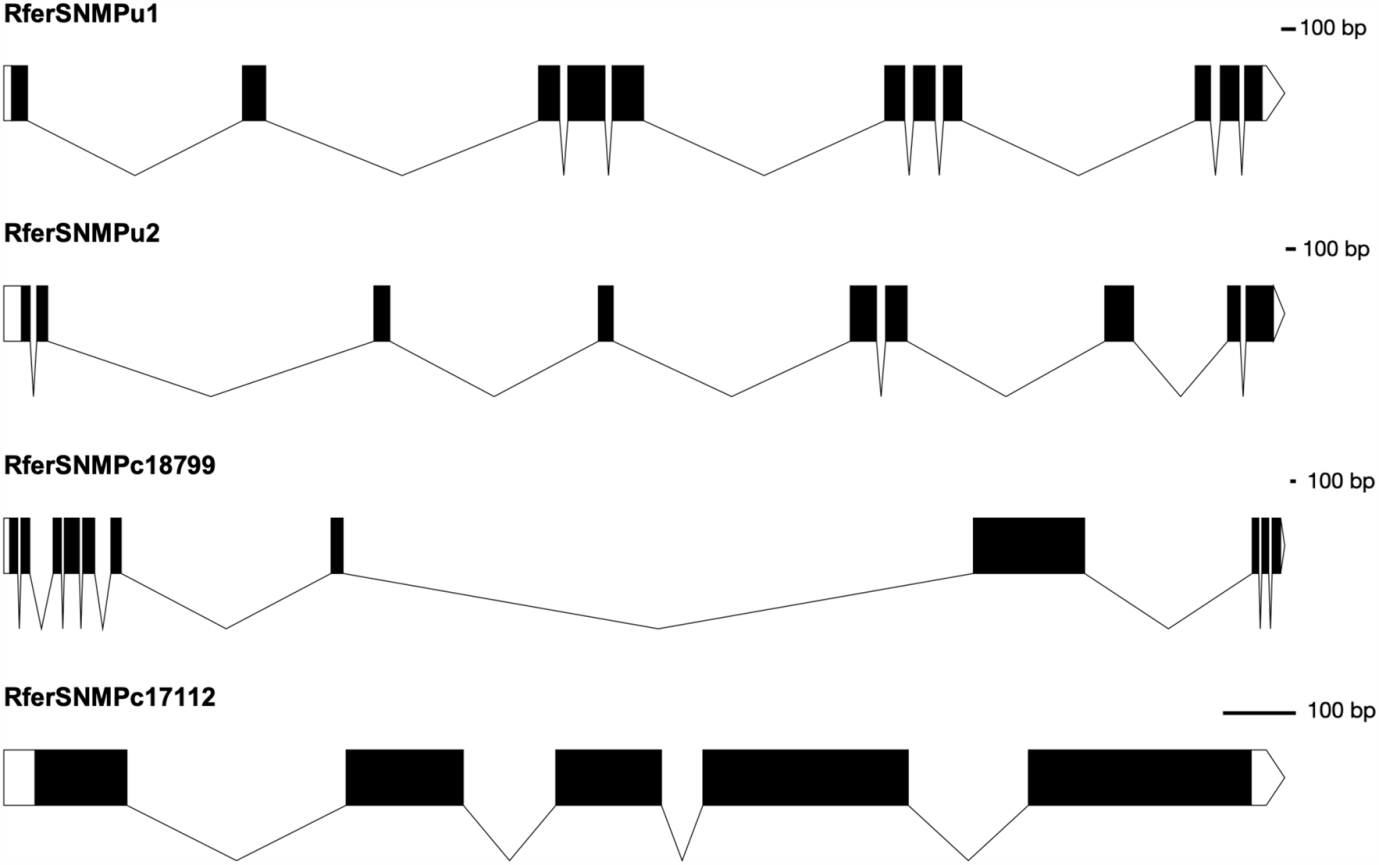
Genome mapping results showing exon-intron organizations of all four RferSNMPs. Exons are represented as thick boxes, and introns as thin lines.

By using NCBI Acc. No. JAACXV010014332 locus_tag = “GWI33_018522” (complement 79632.90682) (scaffold_66120), we annotated a deduced amino acid sequence of the *RferSNMPu1* fragment that was identical to *RferSNMPu1*. The functional *RferSNMPu1* gene length mapped was 11051 bp in scaffold_66120 (Fig. 2, Supplementary Tables S4, S5). The genomic organization of *RferSNMPu1* and *RferSNMPu2* (locus_tag = “GWI33_018521”) revealed that they were distributed across the same scaffold_66120 in the *R. ferrugineus* genome, indicating both SNMPs’ possible emergence through tandem duplication (Supplementary Tables S4, S5). Similarly, *RferSNMPc18799* and *RferSNMPc17112* were also distributed across the same scaffold_66401 (NCBI accession: JAACXV010014582 locus_tag = “GWI33_020629 and GWI33_020630”) in the *R. ferrugineus* genome (Supplementary Tables S4, S5). Basic information about the RferSNMPs male and female mapped reads with the ratio of unique exon and intron reads are provided in Supplementary Tables S4, S5. *RferSNMPu1* and *RferSNMPc18799* contained eleven exons, whereas *RferSNMPu2* and *RferSNMPc17112* had nine and five exons, respectively (Supplementary Tables S4, S5).

### RferSNMP phylogeny and tissue-specific expression analysis

**Figure 3:**
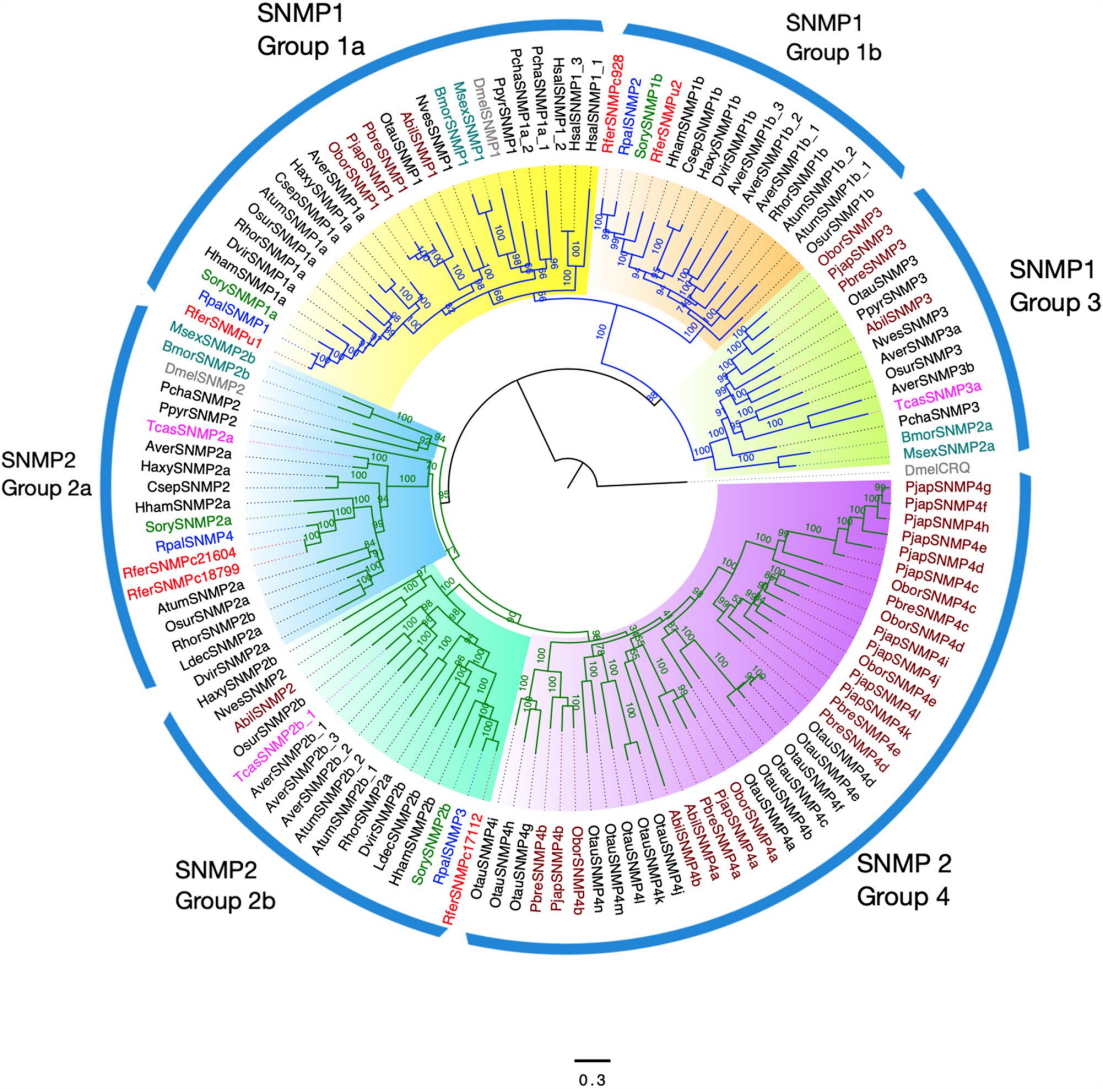
Maximum likelihood consensus tree of sensory neuron membrane proteins (SNMPs) from Coleoptera. The tree was constructed by amino acid sequences of SNMPs from *Rhynchophorus ferrugineus:* Rfer (red), *Rhynchophorus palmarum:* Rpal (blue), *Sitophilus oryzae*: Sory (green), and Scarabaeidae beetles (brown), lepidopterans (dark green), *Drosophila melanogaster*, Dmel (silver) and other Coleopterans (black). The tree was rooted to *D. melanogaster Crq* protein. Branch values represent bootstrap values. Node colors blue and green represent the SNMP1 and SNMP2 classes of proteins, respectively. Individual Groups within each SNMP class are marked and highlighted with colors: Group 1a (yellow), Group 1b (orange), Group 3 (green), Group 2a (blue), Group 2b (cyan) and Group 4 (purple). The scale bar represents amino acid substitutions per site.

The maximum likelihood phylogenetic tree of SNMPs from closely related insect groups rooted with an outgroup non-SNMP *Crq* protein revealed two distinct families of SNMPs, viz*.*, SNMP1 and SNMP2 (Fig. 3). Based on the analysis, we classified two *R. ferrugineus* SNMPs, *RferSNMPu1*, *RferSNMPu2* (or *RferSNMPc928*), as SNMP1 and two RferSNMPs, *RferSNMPc17112*, *RferSNMPc18799* (or *RferSNMPc21604*) as SNMP2 proteins. Based on the phylogeny, SNMPs were further grouped into sub-classes^23,39^ as *RferSNMPu1* and *RferSNMPu2* belonged to SNMP1a and 1b sub-groups, respectively. However, no candidates were identified in SNMP3 and SNMP4 (Scarabaeidae-specific) and SNMP2a subfamilies, as both the SNMP2s in RPW, *RferSNMPc17112*, *RferSNMPc18799* (or *RferSNMPc21604*) belonged to SNMP2a (Fig. 3).

**Figure 4:**
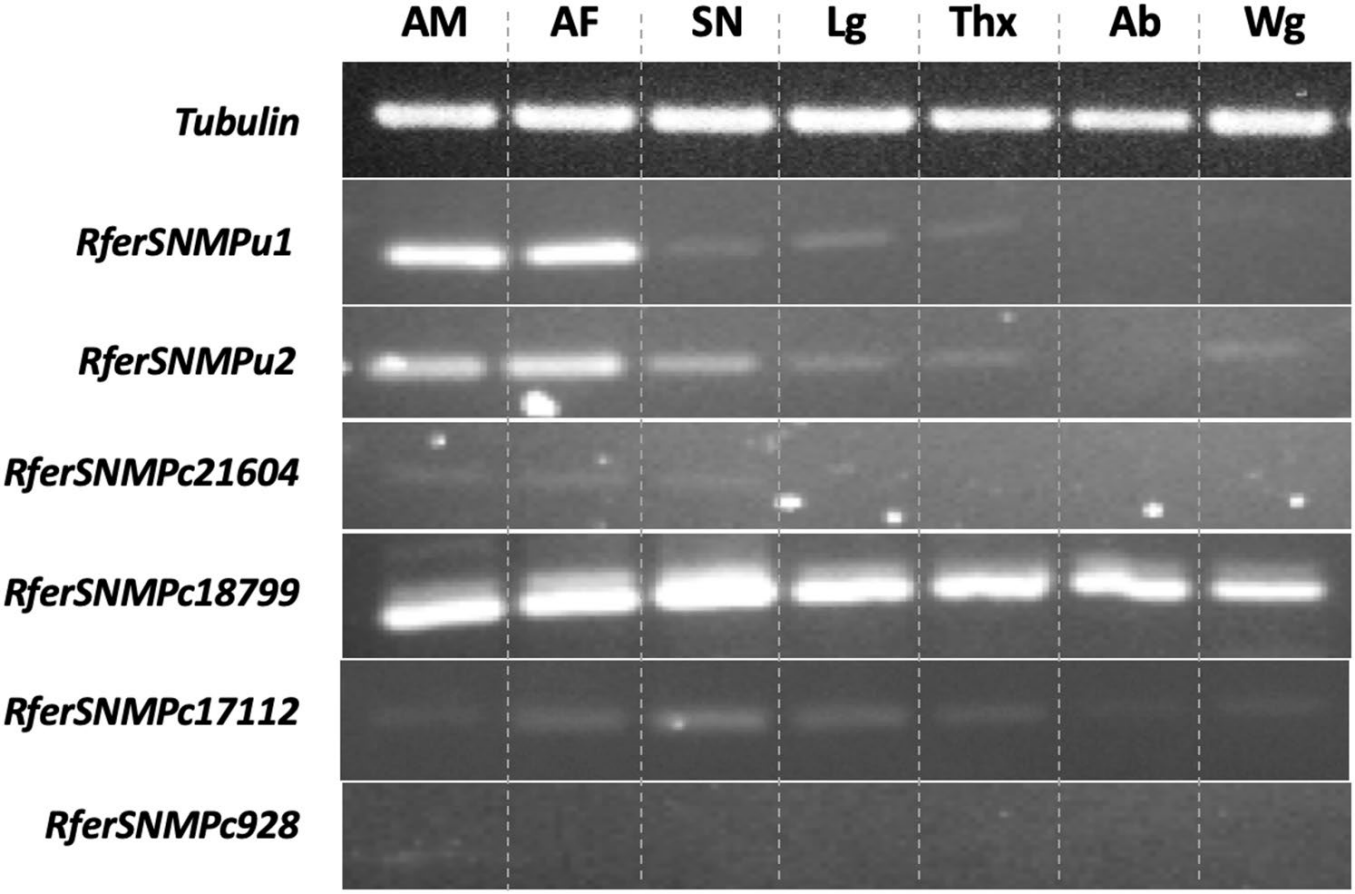
Tissue-specific expression analysis of six SNMPs identified from *Rhynchophorus ferrugineus*^33^. Tissues used are indicated as AM (male antennae), AF (female antennae), SN (male snout), Lg (male legs), Thx (male thorax), Ab (male abdomen), and Wg (male wings). The Tubulin gene was used as a control. Primer details are provided in Table S1.

All six RferSNMPs^33^, including the *RferSNMPc21604* and *RferSNMPc928*, were analyzed for tissue-specific expression using antennae (male), antennae (female), snout (male), legs (male), thorax (male), abdomen (male), and wings (male) (Fig. 4, Figure S1). The results indicated antenna-enriched expression of *RferSNMPu1* and *RferSNMPu2*, whereas *RferSNMPc18799* was ubiquitously expressed in all tissues compared. *RferSNMPc17112* had a moderate antenna-enriched expression, and *RferSNMPc21604* had a substile expression in the snout, whereas *RferSNMPc928* was not detected in any of the analyzed tissues (Fig. 4).

### Pheromone pre-exposure experiments and relative quantification of RferSNMPs expression

**Figure 5:**
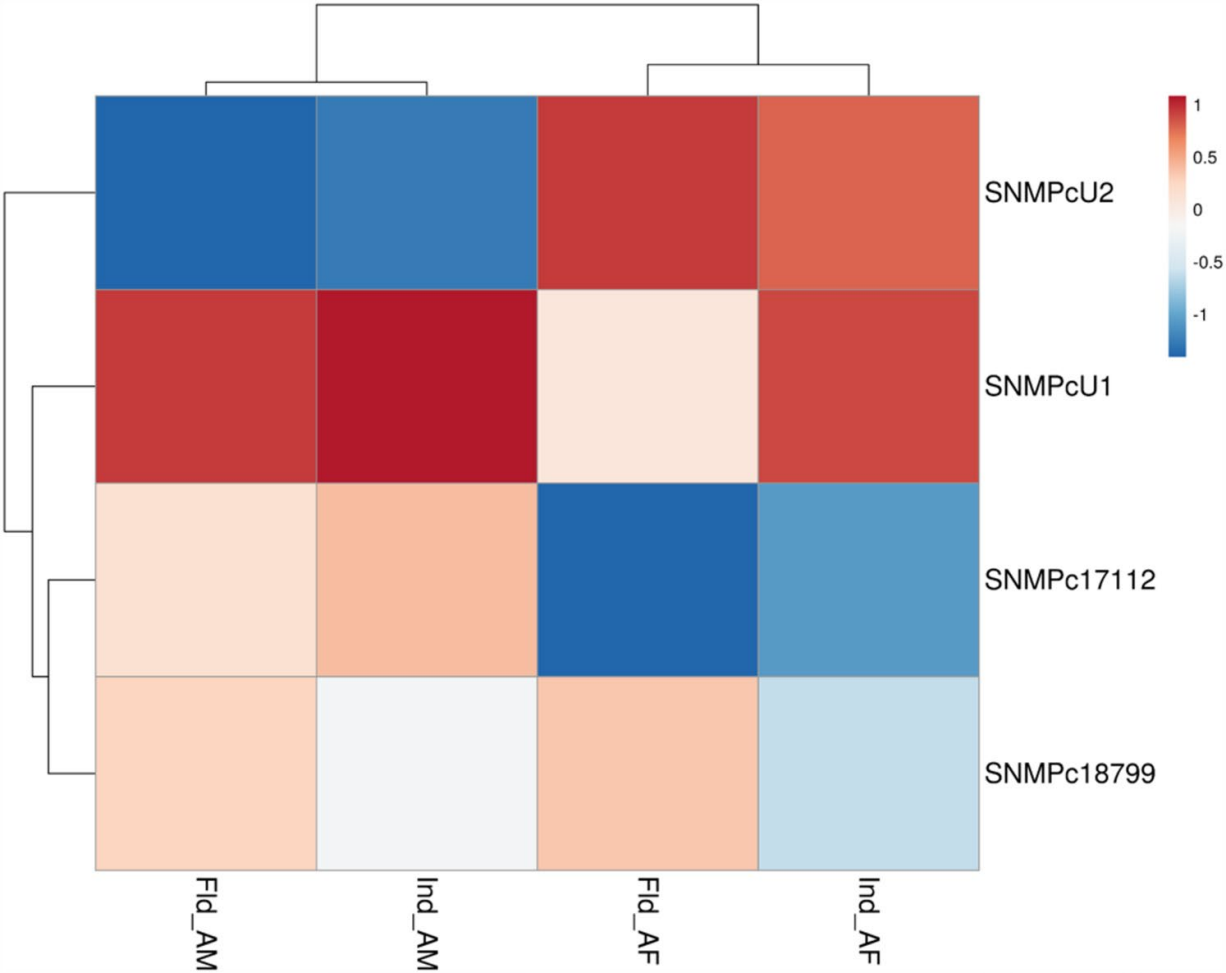
Heatmap showing changes in RferSNMP expression levels in pre-exposed and field-collected RPW adults compared with the RferSNMP respective expression in nonexposed animals from the laboratory colony. The data represents log-transformed 2^−ΔΔCT^ values measured by RT-qPCR. The heatmap colours represent the expression level from highest (blood red) to lowest (dark blue). Ind, insects pre-exposed with a commercial aggregation pheromone; Fld, insects collected from a date palm field in Saudi Arabia—AM, antenna male; and AF, antenna female.

To determine potential RferSNMPs associated with the detection of the aggregation pheromone, we subsequently assessed if there was an increase in the expression levels of any RferSNMP genes after the insect’s exposure to a synthetic pheromone blend. The changes in expression patterns of the four SNMPs expressed in the antennae were relatively insignificant in the group exposed to pheromones compared to the control group in the laboratory setting. Most SNMPs exhibited decreased expression levels compared to nonexposed RPW controls in the laboratory-reared samples. The expression levels ranged from 0.09 to 1.42 of average ∆Ct values (normalized with *Tubulin* and *β-actin* gene expression). Among the targeted genes, *RferSNMPu1* showed slightly higher expression than others, followed by *RferSNMPu2* (Fig. 5). Interestingly, *RferSNMPu1* demonstrated a slightly higher expression in RPWs pre-exposed to pheromones compared to field-caught antenna female samples. However, lower expression levels were observed in field-caught antenna males and pheromone-pre-exposed male RPWs (Fig. 5). Based on the expression analysis using different approaches (qRT-PCRs, pheromone exposure experiments, and TPM), we selected *RferSNMPu1* as a potential candidate for functional analysis.

### RferSNMPu1 structural and in vivo functional analysis

**Figure 6:**
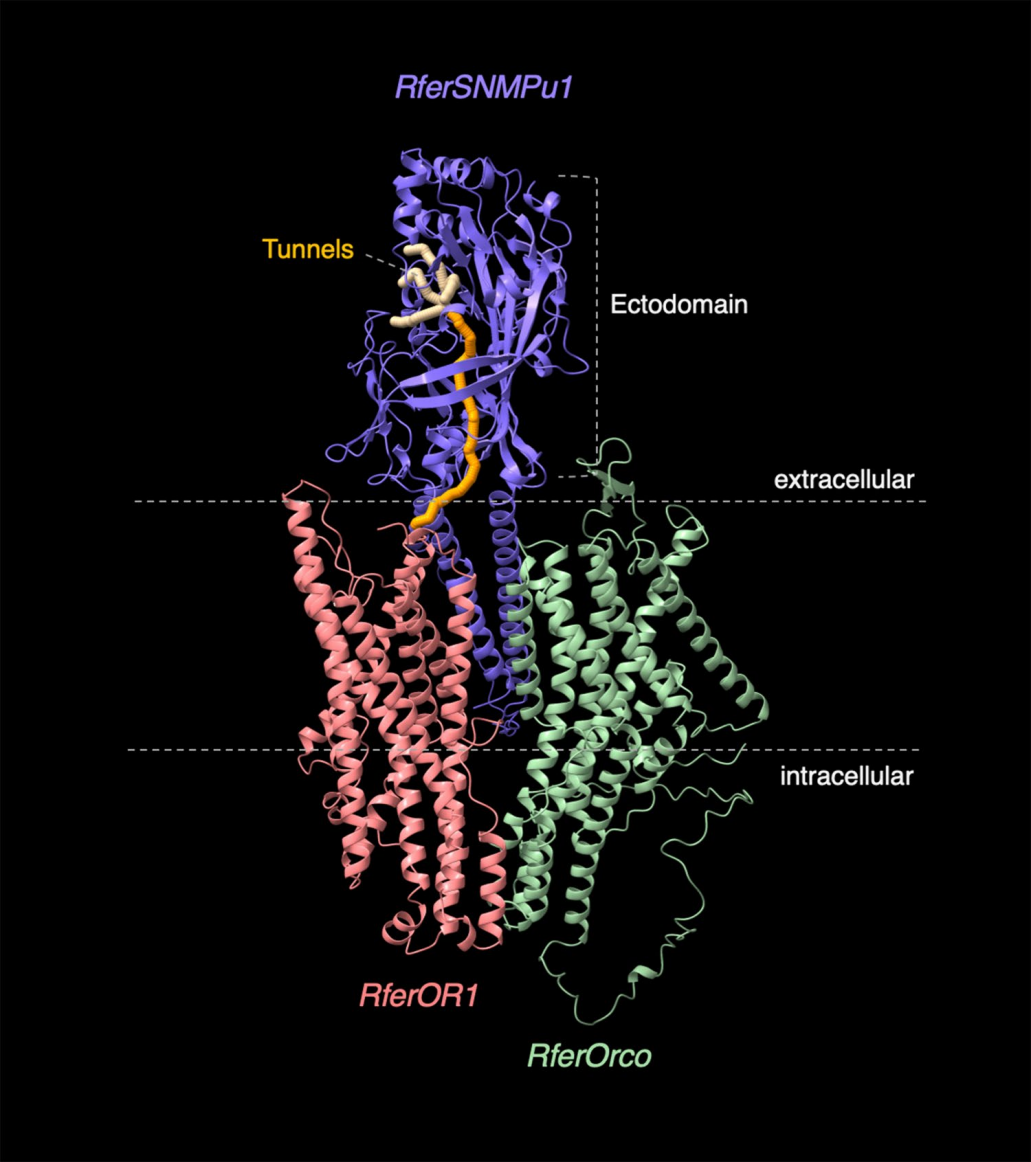
Predicted 3D Structural model of *RferSNMPu1* aligned with monomers of *RferOR1* and *RferOrco*. The identified tunnels within the ectodomain were colored (yellow), with the longest one being ‘orange’ and marked at the tunnel opening. Neuronal membrane boundaries and ectodomain are marked with dashed lines.

Taking the antennal-enriched and high expression level of *RferSNMPu1* and moderate induction of RferSNMP upon pheromone exposure together, we selected it as the most promising SNMP for structural and functional characterization. Based on blastx searches, *RferSNMPu1* shares 74.85% aa sequence identity with *Sitophilus oryzae* SNMP1 (GenBank Acc no. QEX07999.1). The structural elucidation allowed us to explore the structure-function relationship of *RferSNMPu1*. The basic structure of SNMP1 proteins includes two transmembrane domains separated by an ectodomain and flanked by short N- and C-terminal tails, as elucidated first in *Drosophila*^32^. *RferSNMPu1* has been identified as a membrane protein with 544 amino acids in length, and the structural predictions had high accuracy with a pLDDT score of 90.1 for the best-ranked model. We identified the ectodomain, positioned from 27P to 449 K, and the two transmembrane regions predicted were positioned from 7L to 26F and 450I to 472L, respectively. The ectodomain has been proved to be functionally important by mutation analysis in *Drosophila*^32^. The two cytosolic tails were predicted to be positioned as 1 M to 6 K at the N-terminal and 473E to 544S at the C-terminal (Fig. 6, Figure S2). The multimeric model generated with monomers of *RferSNMPu1, RferOR1,* and *RferOrco* with pTM score of 0.563 allowed us to understand better the alignment of these three proteins in the membrane of ORNs (Fig. 6). Further, we predicted catalytic pockets in *RferSNMPu1* and picked the top 10 tunnels based on their bottleneck radius. Interestingly, the longest tunnel (tunnel 9), with 1.5 Å in bottleneck radius and 61.5 Å in length (Figure S3), originated from *RferSNMPu1* ectodomain ends at the opening of *RferOR1* in the multimeric model showcasing structural alignment (Fig. 6). This highlights the potential role of *RferSNMPu1* in transferring pheromone to its particular receptor.

**Figure 7:**
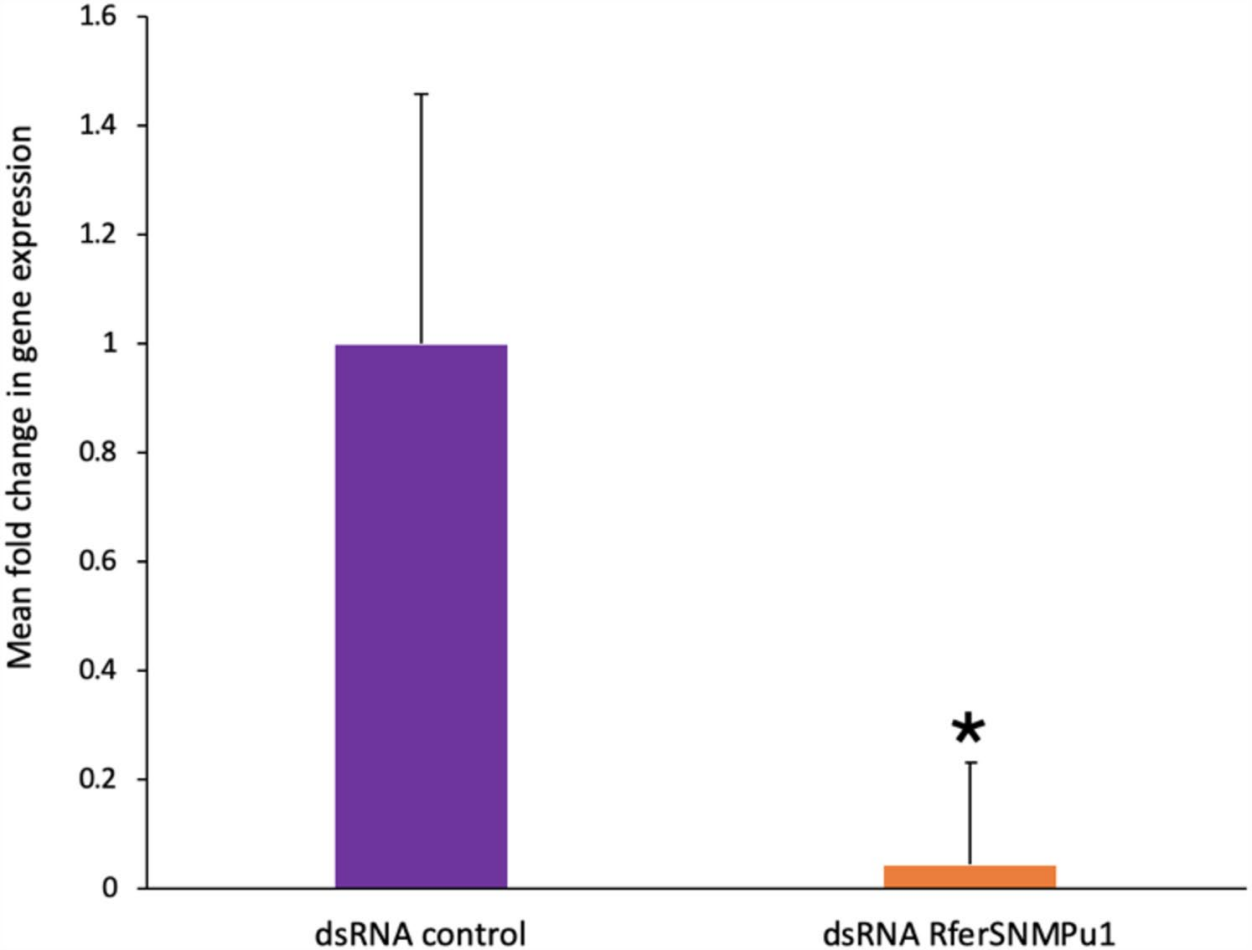
RNA interference-based silencing of *RferSNMPu1* in RPWs. Mean fold change in *RferSNMPu1* expression (2^−ΔΔCT^ values) was compared between dsRNA control and dsRNA RferSNMPu1 injected RPWs. (*) indicates a significant difference between the two groups with alpha level *P* < 0.05. (*P* = 0.040). Error bars represent SEM.

**Figure 8:**
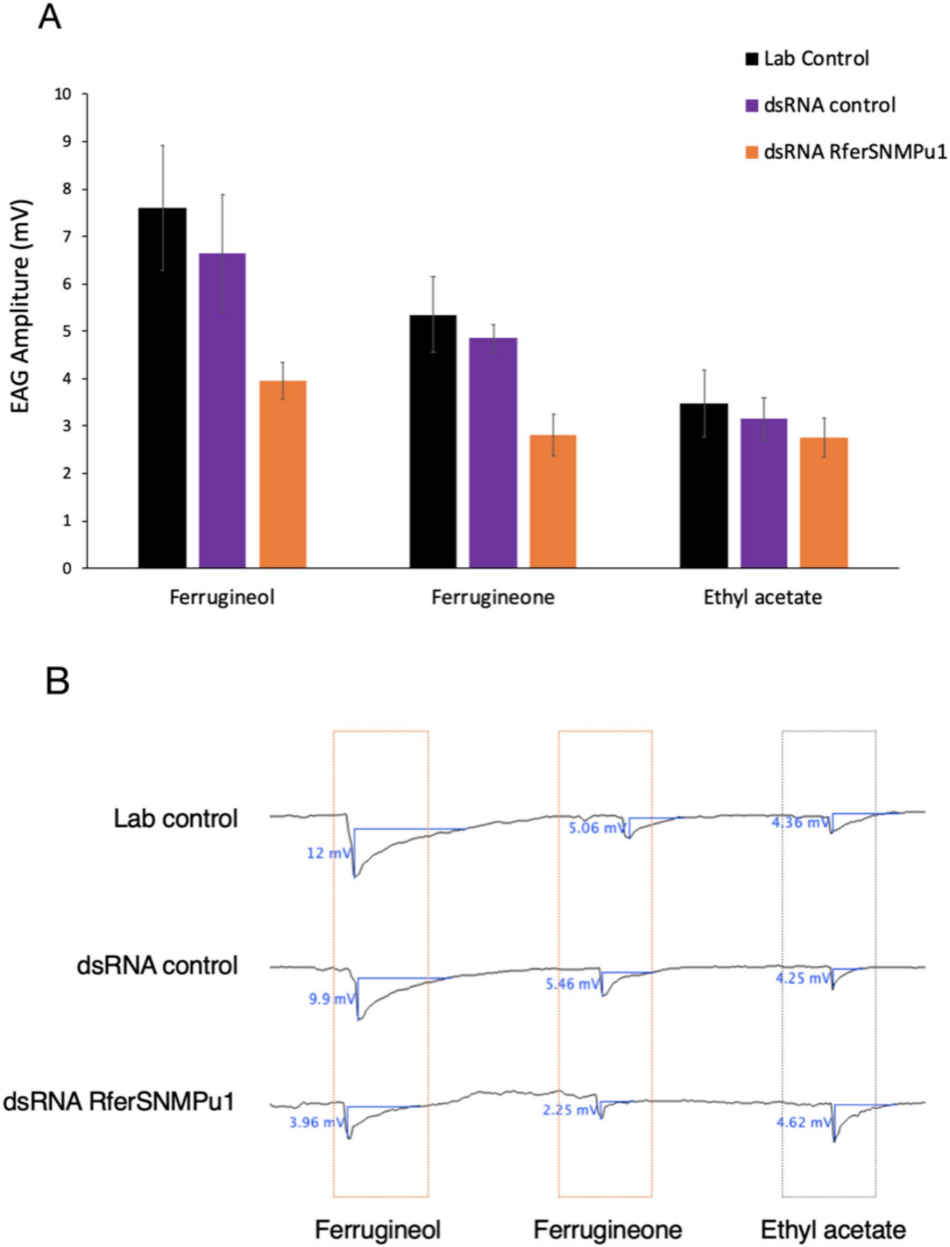
(**A**) Effect of *RferSNMPu1* gene silencing in the electroantennogram responses (EAG) of *Rhynchophorus ferrugineus* in three experimental groups: Lab control, dsRNA control, and dsRNA RferSNMPu1 against three stimuli ferrugineol, ferrugineone, and ethyl acetate. EAG responses significantly differ from the control groups marked with (*). Error bars represent SEM. (**B**) Representative EAG responses from lab control, dsRNA control, and dsRNA RferSNMPu1 group of insects with respective stimuli Pheromones: ferrugineol and ferrugineone and kairomone: ethyl acetate. Measurements were performed at 10 mV and 15 s intervals.

The relative expression of *RferSNMPu1* upon gene silencing was validated by comparing it to the dsRNA control injected. The percentage of knockdown in the test RPWs (dsRNA RferSNMPu1 injected) adults was estimated as 95.47% compared to the control (dsRNA control injected) group (*p-*value = 0.040) (Fig. 7). Electroantennograms were recorded from the antennae of the control and experimental group adult RPWs at 21 days of emergence. The antennal response to three different stimuli, ferrugineol and ferrugineone (pheromone component), and ethyl acetate (kairomone), were recorded from each with normal laboratory-reared RPWs (‘lab control’) and compared to dsRNA RferSNMPu1 and dsRNA control injected RPWs. The control groups, viz., dsRNA control and lab control, showed no significant differences in antennal responses between ferrugineol, ferrugineone, and ethyl acetate with observed *p-*values of 0.331, 0.586, and 0.706, respectively (Fig. 8A, Table S6). The dsRNA RferSNMPu1 injected adults showed a significant reduction in antennal responses compared to both control groups (lab control and dsRNA control) when stimulated with ferrugineol and ferrugineone and not with ethyl acetate (Fig. 8A,B). The response to ferrugineol was significantly reduced in dsRNA RferSNMPu1 injected adults with *p*-values 0.001 and 0.020, respectively, for lab control and dsRNA control (Table S6). Similarly, ferrugineone response was significantly reduced compared to both control groups, with *p*-values of 0.002 and 0.014. However, for ethyl acetate stimuli, no significant differences were observed between the three groups (Table S6).

## Discussion

The current research explores the essential role of sensory neuron membrane proteins (SNMPs) in red palm weevil pheromone communication to identify potential pest control targets. SNMPs belong to a class of CD36 superfamily of proteins found in vertebrates and are identified in insect antennal sensilla lymph as one of the abundant proteins secreted by OSNs and supporting cells^20,40^. Six SNMPs were previously reported in the RPW antennal transcriptome^33^, and two SNMPs were found to be identical based on genome-wide analysis, thereby confirming four SNMPs in RPW antennae. The number of SNMPs was similar to that observed in other insect lineages, including dipterans, lepidopterans, and weevils, except for the lineage-specific expansion found in the Scarabaeidae beetles^39^. Based on phylogeny, we classified RferSNMPs into the two major insect SNMP classes, SNMP1 and SNMP2^20,26^, and identified two representing candidates in each group. The identified SNMPs were orthologous to the ones reported in other weevils^53^.

As the function of SNMPs is primarily attributed to their localized expression, we tested the tissue-specific expression patterns of each RferSNMP. No sex-specific antennal expression was observed in any of the RferSNMPs tested, similar to the expression pattern observed for *RferOR1*^10^, a key pheromone receptor with which *RferSNMPu1* is most likely to interact. We mapped *RferSNMPu1* in the antennae of both sexes, and we did not find any differential expression in both sexes. Similarly, there is no sex-biased expression of the *RferOR1*^10^, coreceptor^12^, and *RferSNMPu1* (current study) in male and female antennae also support the idea that the *RferSNMPu1* might have a possible role pheromone detection. Since aggregation pheromone attracts both male and female RPWs, it makes sense that other olfactory genes (*RferOR1* and *RferOrco*) would have no sex-biased expression^10^, shed light on the possible interaction of *RferOR1* and *RferSNMPu1* in pheromone detection. Besides, we found an antennal-enriched expression of *RferSNMPu1*, similar to *RferOR1*, which was later found to be expressed only in the RPW antenna^10^. Conversely, *RferSNMPu2* found less expressed than *RferSNMPu1,* and had an expression pattern matching SNMP1s, suspecting its potential role in the pheromone-clearing process^25^. In contrast, one SNMP2 protein, *RferSNMPc18799*, showed ubiquitous expression; the other, *RferSNMPc17112*, showed subtle expression in various tissues. Interestingly, since almost half of RferORs are expressed throughout the body^10^, it is very likely that the SNMPs that are associated with ORs are expressed throughout the body. However, only a limited number of RPW ORs have been characterized to make a functional comparison as interacting with RferSNMPs^10^. As we were aimed at antenna-enriched SNMPs with a potential role in pheromone communication, we quantified the two highly expressing SNMPs from each class, SNMP1 and SNMP2, in the male and female antennal tissues. After that, we targeted the highly and uniquely expressed SNMP (*RferSNMPu1*) in the antennae for further studies similar to the pheromone receptor (*RferOR1*), which is highly expressed in *R. ferrugineus* and hence will have a potential role in pheromone detection. All identified RferSNMPs appeared with their rostrum (snout) counterpart (i.e., *RferS_SNMP2a, RferS_SNMP2b, RferS_SNMP_U2*, and *RferS_SNMP_U1* with 100, 99.7, 96.1, and 99.4% of sequence identity, respectively), as reported recently^54^. The manual genome^55^ annotations revealed the two SNMPs, *RferSNMPu1* and *RferSNMPu2*, in scaffold 66120 (locus tag GWI33_018522 and GWI33_018521, respectively), and the other two, *RferSNMPc18799* and *RferSNMPc17112,* in scaffold 66401 (locus tag GWI33_020629 and GWI33_020630, respectively). All four SNMP sequences hit different genomic positions, meaning they could be independent genes.

Additionally, the pheromone induction experiments were performed to assess the changes in the expression of these genes in response to pheromone. We found *RferSNMPu1* transcript upregulation upon exposure to pheromone stimuli in both male and female RPW adults. Based on recent studies on expression analysis of other key olfactory genes, viz., Orco^12^ and pheromone receptor^10^, the higher expression reported in 5–20 days compared to newly emerged adults. Besides, a relevant study demonstrated that the release of the palm weevil aggregation pheromone starts approximately 10 min after the insect detects palm tree emitted volatile such as ethyl acetate and continues for several hours^56^. Considering the RPW life span is over 60 days^57^, the olfactory protein expression would be higher during earlier stages^10,12^; we assume that *RferSNMPu1* expression would be similar to that of other olfactory proteins involved in pheromone detection. Nevertheless, both *RferSNMPu1* and pheromone receptor^10^ are transmembrane proteins; hence, even pheromone exposure did not result in a significant expression variation in 5 days RPW adults, whereas secretory proteins like OBPs show significant variations in expression according to age^9^. However, the constitutive expression of olfactory proteins, including SNMP1, may likely be determined by age; hence, an appropriate further comparison of SNMP1 expression level is recommended to verify on same-age animals in future studies.

For *RferSNMPu2,* we observed changes in the expression between male and female samples. Such differences are reported in other coleopteran SNMPs, like in *Rhaphuma horsfieldi,* based on transcriptome analysis^39^. The RPW transcriptome-based expression analysis revealed no difference in male and female RferSNMPs from lab and field-collected samples. However, SNMP1 proteins were more abundant than SNMP2 proteins in the antennal transcriptomes, similar to the pattern observed in other coleopterans like *R. horsfieldi*^39^ and *Speonomus longicornis*^58^. The classification and expression analysis concluded the identification of *RferSNMPu1*, a relatively abundant SNMP1 protein, as a potential candidate for functional studies.

The structural elucidation using a machine learning-based algorithm allowed us to explore the key functional domains in *RferSNMPu1*. Although decades have passed since the original discovery of SNMPs in moths as a ~ 69 kDa protein^17^, the structure-to-function relationships were explored only recently. The basic structure of SNMP1 proteins includes two transmembrane domains, separated by an ectodomain and flanked by short N- and C-terminal tails, as elucidated first in *Drosophila*^32^. The lack of structural studies in SNMPs is still a limitation for in silico modelling. However, the availability of CD36 protein crystallographic structures from higher mammalian orders^59^ provided a trustworthy prediction of the ectodomain and the two transmembrane domains in *RferSNMPu1* assisted by deep-learning algorithms^45^. However, the prediction of a long intracellular cytosolic tail at the C-terminal was less accurate (Figure S4) as predictions are based on sequence alignments. The structure-function relationships explored using mutation analysis of ectodomain in *Drosophila* clearly defined the importance of ectodomain, which acts as a tunnel to release and transfer pheromone molecule from OBP to the ligand-binding site within the OR/Orco complex^32^. We analyzed the ectodomain and predicted the tunnels within the ectodomain, which allows the passage of RPW pheromones. The predicted longest tunnel in *RferSNMPu1* originated from the ectodomain opening and ended close to proximity with the pheromone receptor *RferOR1,* which underlines the tunnelling mechanism proposed in *Drosophila* pheromone detection^32^. The long C-terminal cytosolic tail in *RferSNMPu1* could indicate a potential role of this structure in intracellular signaling cascades. Still, the functional studies on short N-and C-terminal cytosolic tails in *Drosophila* SNMPs contrast this possibility^32^. The cytosolic tails could involve in the formation of multimeric structures; however, we currently lack such information at homo/hetro tetrameric levels due the lack of crystallographic structures. The current knowledge of Coleoptern OR-Orco alignment in the ORN membrane is minimal. However, we propose that the two characterized RPW ORN membrane proteins *RferOrco* and *RferOR1* may likely be interacting with *RferSNMPu1* based on their proven role in RPW pheromone detection.

Gene silencing approaches have been widely used to in vivo functionally characterize and validate potential candidate genes in insects, with a high success rate, especially in the Coleopterans^50,60^. In *R. ferrugineus,* such approaches have already been key to identifying and characterizing key olfactory proteins, like odorant receptor coreceptor^12^, pheromone binding proteins^9^, and pheromone receptor^10^. Hence, we explored the other olfactory protein elements in lipophilic pheromone communication in insects, the SNMPs. This makes sense as many insect pheromones are lipid-derived, and two different SNMP classes play two distinct roles proposed as SNMP1 in pheromone binding and SNMP2 in pheromone clearance. We successfully silenced *RferSNMPu1* using RNAi and tested the odour response in the gene-silenced RPWs. EAG has been established for a long time as a standard technique to assess neuronal responses to odors directly from the antennae. This technique has been well-established in *R. ferrugineus* and has been widely used as a standard to determine the silencing of olfactory-related proteins^9,10,12^. We found that SNMP1 silencing reduced the responses to pheromone components ferrugineol and ferrugineone in adult weevils, whereas it normally responded to ethyl acetate compared to respective controls. These results indicate the proposed function of the SNMP1 class of protein in detecting RPW pheromone components. In *Drosophila*, SNMPs are found exclusively essential in pheromone-sensitive neurons for cVA sensitivity but not for the sensitivity to general odorants^21^. The co-expression studies in locust *Schistocerca gregaria*, propose the involvement of SNMP1s in the functioning of only a limited number of ORs (33 out of 83) expressed in OSNs^61^. The in vitro functional expression studies in *Heliothis virescens* have also shown that SNMP1 is essential for detecting pheromone compounds by ORs, compared to the cells without SNMP1 and cells expressing SNMP2^30^. Nonetheless, within such heterologous expression systems, responses may also be elicited in the absence of another crucial component, Orco, thereby casting doubt on the role of SNMPs in moths. Recently, the double RNAi knockdown experiments using SNMP1 and OR in *Bombyx mori* followed by behavioral assays have proved the essential role of SNMP in moth pheromone detection^62^. However, these in vivo knockdown studies have limitations in understanding the interactions between SNMP1 and other key receptor proteins in the membrane. Heterologous expression systems like yeast-two hybrid have successfully demonstrated the interactions between SNMP and other key proteins, OR and Orco in *Bombyx mori*^62^. However, in RPWs, the essential role of Orco^12^ in pheromone detection has already been reported, along with identifying the key pheromone receptor, *RferOR1*^10^. Interestingly, *RferOR1* was transgenically expressed in the trichoid sensilla of *Drosophila* that carry SNMPs, indicating that SNMPs are involved in RPW pheromone communication^10^. Our finding concludes that *RferSNMPu1* may likely be essential in RPW pheromone communication. However, the interactions between *RferSNMPu1* and other key membrane receptors (*RferOR1*) have yet to be explored, suggesting further functional studies.

To summarize, we identified, classified, and genome-mapped the four SNMPs in the RPW antenna, one of the key pests of palm trees around the globe. We identified a key member of the SNMP family, *RferSNMPu1*, with a potential role in pheromone detection, as a key target for olfactory disruption-based pest control strategies. Our in vivo functional analysis using RNAi-based silencing of *RferSNMPu1* exclusively impaired the detection of pheromone components in the weevils. It shed light on the possible role of *RferSNMPu1* in RPW pheromone detection. These findings also serve as an initial attempt to understand the SNMP family with a less explored functional role in coleopterans and other insects.

### Supplementary Information

Supplementary Information 1.

Supplementary Tables.

## Data Availability

The datasets generated and/or analyzed during the current study are available in the NCBI repository under BioProject PRJNA275430 and SRA accession numbers: SRR22098129; SRR22098128, SRR22098127 and SRR22098126, and *R. ferrugineus* genome (GenBank: GCA_014462685).
