# Supplementary material for: Silencing sensory neuron membrane protein RferSNMPu1 impairs pheromone detection in the invasive Asian Palm Weevil
Source: Sci Rep. 2024 Jul 17;14:16541. doi: 10.1038/s41598-024-67309-x (PMC11254914; doi:10.1038/s41598-024-67309-x)
Supplement: Supplementary file 1 — Supplementary Information 1. [file 41598_2024_67309_MOESM1_ESM.docx]

**Supplementary information for**

Silencing sensory neuron membrane protein *RferSNMPu1* impairs pheromone detection in the invasive Asian Palm Weevil

Jibin Johny^1,2,3^, Mohammad Nihad^1^, Hattan A. Alharbi^1^, Mohammed Ali AlSaleh^1^ and Binu Antony^1,3*^

*^1^Department of Plant Protection, Chemical Ecology and Functional Genomics Lab, College of Food and Agricultural Sciences,* *King Saud University, 11451 Riyadh, Saudi Arabia.*

*^2^*Present address: *Faculty of Forestry and Wood Sciences, Czech University of Life Sciences, Prague, Czechia.*

^3^These authors contributed equally: Jibin Johny and Binu Antony

**Corresponding author: E-mail: bantony@ksu.edu.sa; Phone: +966-11-46-93377*

**List of Contents**

**Table S1.** List of primers used in the experiments.

**Table S2.** Mapping the expression level distribution of RferSNMPs, obtained from the transcript quantification of field-collected and lab-reared *R. ferrugineus* - male vs. female.

**Table S3.** Mapping the expression level distribution of RferSNMPs, obtained from the transcript quantification of male and female *R. ferrugineus* – field-collected vs. lab-reared.

**Table S4.** Genome-wide analysis and expression mapping of RferSNMPs on *R. ferrugineus* male antennal transcriptome. (Microsoft Excel Table S4 and S5_sheet2)

**Table S5.** Genome-wide analysis and expression profiling of RferSNMPs on *R. ferrugineus* female antennal transcriptome. (Microsoft Excel Table S4 and S5_sheet3)

**Table S6:** Statistics data on EAG measurements. EAG responses from each group of weevils were analyzed by the one-way ANOVA with LSD, and *p-values* were compared within groups and provided in the table (α- level of significance *p* < 0.05). Values with significant differences were highlighted in bold.

**Figure S1.** The original gel images of RferSNMPs in tissue specific expression studies.

**Figure S2.** DeepTMHMM transmembrane prediction results of *RferSNMPu1*

**Figure S3.** Predicted top 10 tunnels in the *RferSNMPu1* by Caver Analyst 2.0. Tunnel length in Å and bottleneck radius in Å are plotted in x and y axis respectively.

**Figure S4.** *RferSNMPu1* structure prediction accuracy by Alphafold2: 3D Model ranked 1 is displayed with colours representing pIDDT value. The predicted Local Distance Difference Test (lDDT) score per amino acid position of all five models generated (ranked 1 to 5) are provided in the insight graph.

**Table S1.** List of primers used in the experiments.

| **Primer name** | **Direction** | **Primer sequence in 5’ to 3’ direction** |
| --- | --- | --- |
| RferSNMPc928F | Forward | AGTTCACAGTGGTGCGAAAC |
| RferSNMPc928R | Reverse | TGAGGAAGATGCAGTGACCA |
| RferSNMPc17112F | Forward | TGATCAGCATGACCGCAGAT |
| RferSNMPc17112R | Reverse | GGTTCCGGAGCTCCATCTT |
| RferSNMPc18799F | Forward | TAAGCAAGCGGGTGGAATCA |
| RferSNMPc18799R | Reverse | TACCATCAATGTCCGCCGTT |
| RferSNMPc21604F | Forward | ATTGCAAGAACTTGGACCTT |
| RferSNMPc21604R | Reverse | TCGAGATAGCTTCGACTTGA |
| RferSNMPu1F | Forward | TCGACGGACAACCGAAGATG |
| RferSNMPu1R | Reverse | CTGAGAACACGCTGACTGGT |
| RferSNMPu2F | Forward | GCGCGAAACATGCCTTGAG |
| RferSNMPu2R | Reverse | AAGAGGACCTTCCAGGGAGA |
| RferSNMPu1RiF | Forward | CGAGTGGTTGCGCGTTCTTT |
| RferSNMPu1RiR | Reverse | CGCACCCGGTTTGGTTCTCA |
| RferSNMPu1RiT7F | Forward | TAATACGACTCACTATAGGGCGAGTGGTTGCGCGTTCTTT |
| RferSNMPu1RiT7R | Reverse | TAATACGACTCACTATAGGGCGCACCCGGTTTGGTTCTCA |

**Table S2.** Mapping the expression level distribution of RferSNMPs, obtained from the transcript quantification of field-collected and lab-reared *R. ferrugineus* - male vs. female.

| **SNMPs** | **Locus name** | **Chromosome** | **Region** | **Max group mean** | **Log₂ fold change** | **Fold change** | **P-value** | **FDR**  **P-value** | **Bonferroni** |
| --- | --- | --- | --- | --- | --- | --- | --- | --- | --- |
| *RferSNMPu1* | GWI33_018522 | JAACXV010014332 | Complement (79632..90682) | 232.5553931 | 0.4934044 | 1.407762915 | 0.43086413 | 0.999990455 | 1 |
| *RferSNMPu2* | GWI33_018521 | JAACXV010014332 | Complement (66067..76621) | 252.8014657 | -0.2199092 | -1.164660322 | 0.738887996 | 0.999990455 | 1 |
| *RferSNMPc18799* | GWI33_020629 | JAACXV010014582 | 153154..175891 | 1.326870655 | 0.5634909 | 1.477840807 | 0.516312008 | 0.999990455 | 1 |
| *RferSNMPc17112* | GWI33_020630 | JAACXV010014582 | 181530..183187 | 0.942309615 | 0.6357115 | 1.553703782 | 0.577805413 | 0.999990455 | 1 |

**Table S3.** Mapping the expression level distribution of RferSNMPs, obtained from the transcript quantification of male and female *R. ferrugineus* – field-collected vs. lab-reared.

| **SNMPs** | **Locus name** | **Chromosome** | **Region** | **Max group mean** | **Log₂ fold change** | **Fold change** | **P-value** | **FDR**  **P-value** | **Bonferroni** |
| --- | --- | --- | --- | --- | --- | --- | --- | --- | --- |
| *RferSNMPu1* | GWI33_018522 | JAACXV010014332 | Complement (79632..90682) | 238.8788276 | 0.003150696 | 1.002186282 | 0.99344325 | 0.998791954 | 1 |
| *RferSNMPu2* | GWI33_018521 | JAACXV010014332 | Complement (66067..76621) | 234.4622024 | 0.52586724 | 1.439798819 | 0.16516327 | 0.551223756 | 1 |
| *RferSNMPc18799* | GWI33_020629 | JAACXV010014582 | 153154..175891 | 1.336771422 | 0.048104232 | 1.033905431 | 0.93060877 | 0.984073816 | 1 |
| *RferSNMPc17112* | GWI33_020630 | JAACXV010014582 | 181530..183187 | 0.854454687 | 0.68475324 | 1.607427023 | 0.38259664 | 0.77773365 | 1 |

**Table S6:** Statistics data on EAG measurements. EAG responses from each group of weevils were analyzed by the one-way ANOVA with LSD, and *P* values were compared within groups and provided in the table (α-level of significance *P*<0.05). Values with significant differences were highlighted in bold.

| Variable | (I) Experimental Group | (J) Variables | Mean Difference (I-J) | Std. Error | Significance |
| --- | --- | --- | --- | --- | --- |
|  |  |  |  |  |  |
| **Ferrugineol** | Lab control | dsRNA control | 1.15889 | 1.16850 | .331 |
|  |  | dsRNA RferSNMPu1 | 3.63240^*^ | .92378 | .001 |
|  | dsRNA control | Lab control | -1.15889 | 1.16850 | .331 |
|  |  | dsRNA RferSNMPu1 | 2.47351^*^ | .98868 | .020 |
|  | **dsRNA RferSNMPu1** | Lab control | -3.63240^*^ | .92378 | **.001** |
|  |  | dsRNA control | -2.47351^*^ | .98868 | **.020** |
| **Ferrugineone** | Lab control | dsRNA control | .49589 | .89826 | .586 |
|  |  | dsRNA RferSNMPu1 | 2.51646^*^ | .71014 | .002 |
|  | dsRNA control | Lab control | -.49589 | .89826 | .586 |
|  |  | dsRNA RferSNMPu1 | 2.02056^*^ | .76003 | .014 |
|  | **dsRNA RferSNMPu1** | Lab control | -2.51646^*^ | .71014 | **.002** |
|  |  | dsRNA control | -2.02056^*^ | .76003 | **.014** |
| **Ethyl acetate** | Lab control | dsRNA control | .32904 | .86133 | .706 |
|  |  | dsRNA RferSNMPu1 | .47316 | .68094 | .494 |
|  | dsRNA control | Lab control | -.32904 | .86133 | .706 |
|  |  | dsRNA RferSNMPu1 | .14412 | .72878 | .845 |
|  | dsRNA RferSNMPu1 | Lab control | -.47316 | .68094 | .494 |
|  |  | dsRNA control | -.14412 | .72878 | .845 |

**Figure S1.** Agarose gel images of RferSNMPs reported in tissue specific expression studies.


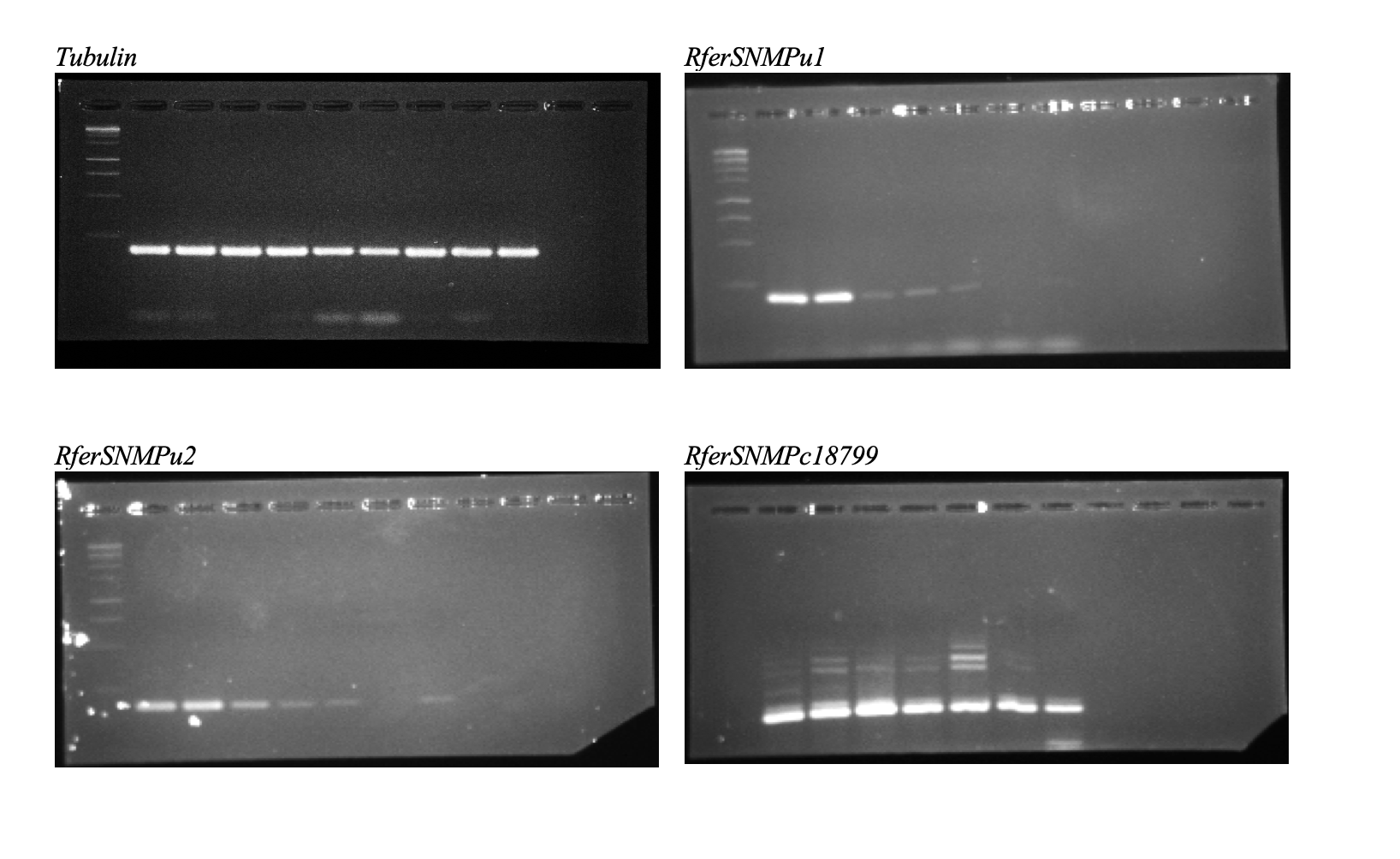


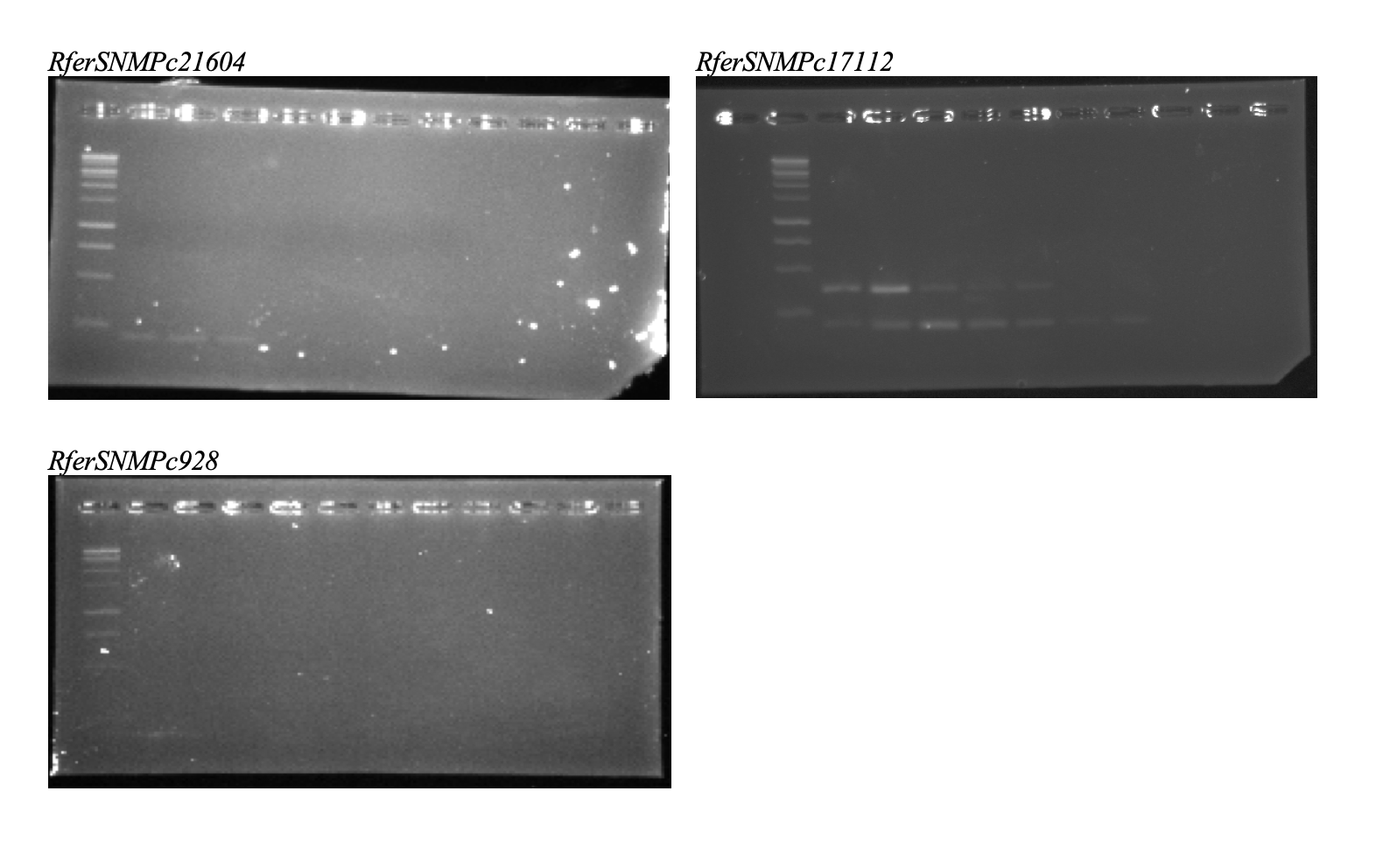


**Figure S2.** DeepTMHMM transmembrane prediction results of RferSNMPu1


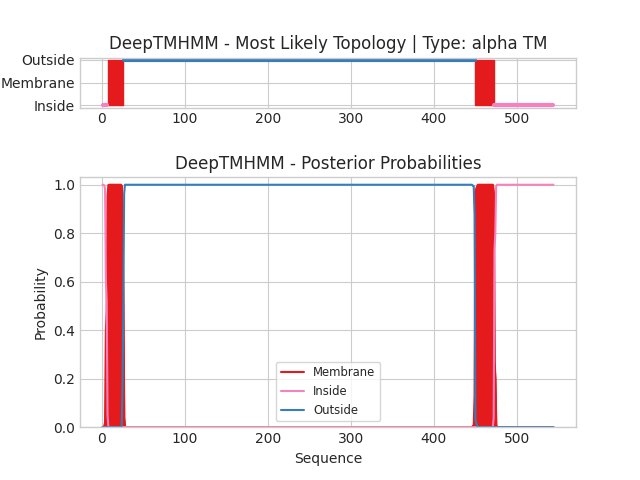


# RferSNMPu1 Length: 544

# RferSNMPu1 Number of predicted TMRs: 2

| RferSNMPu1 | inside | 1 | 6 |
| --- | --- | --- | --- |
| RferSNMPu1 | TMhelix | 7 | 26 |
| RferSNMPu1 | outside | 27 | 449 |
| RferSNMPu1 | TMhelix | 450 | 472 |
| RferSNMPu1 | inside | 473 | 544 |

**Figure S3.** Predicted top 10 tunnels (T1 to T10) in the *RferSNMPu1* by Caver Analyst 2.0. Tunnel length in Å and bottleneck radius in Å are plotted in x and y axis respectively.

***
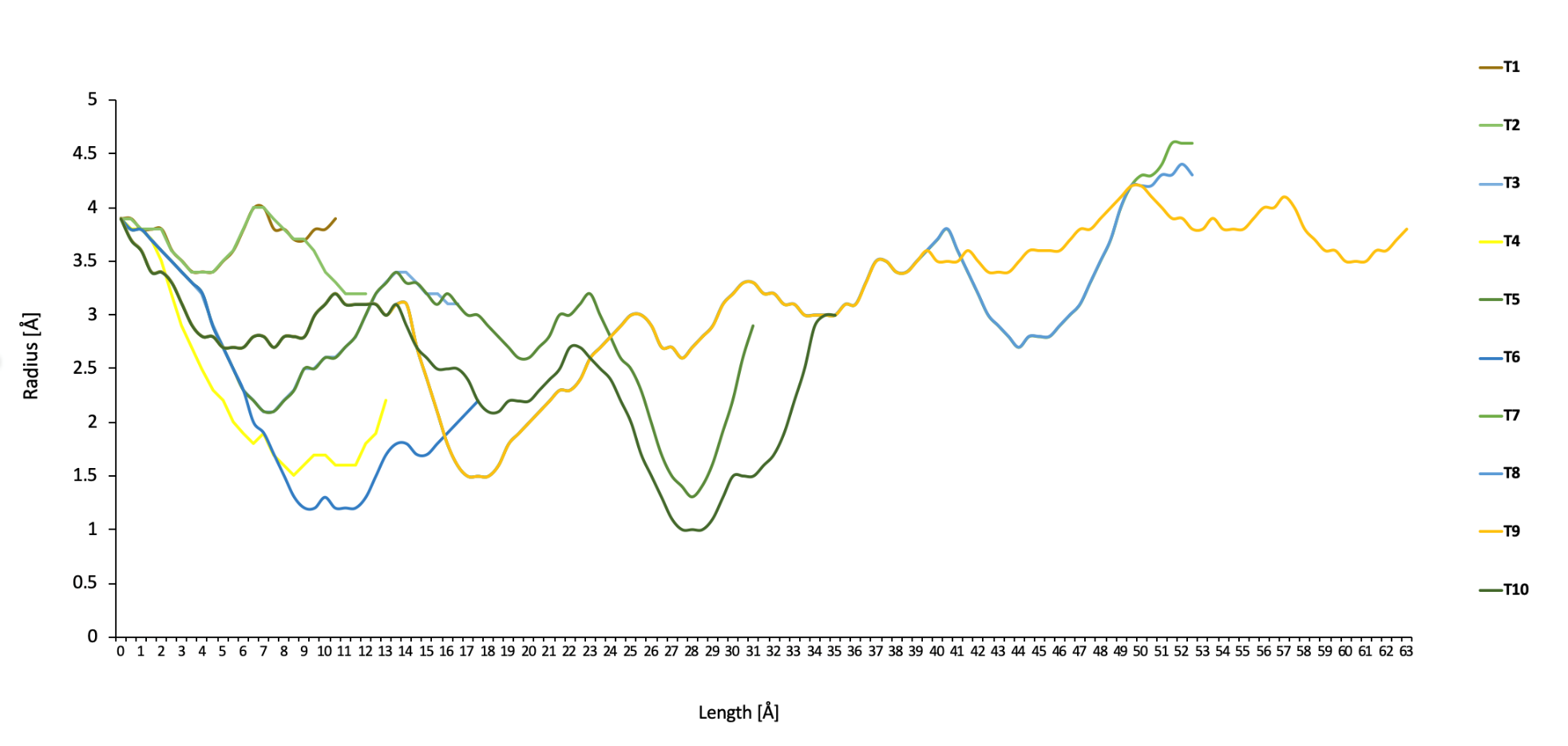
***

**Figure S4.** *RferSNMPu1* structure prediction accuracy by Alphafold2: 3D Model ranked 1 is displayed with colors representing pIDDT value. The predicted Local Distance Difference Test (lDDT) score per amino acid position of all five models generated (ranked 1 to 5) provided as insight graph.

**
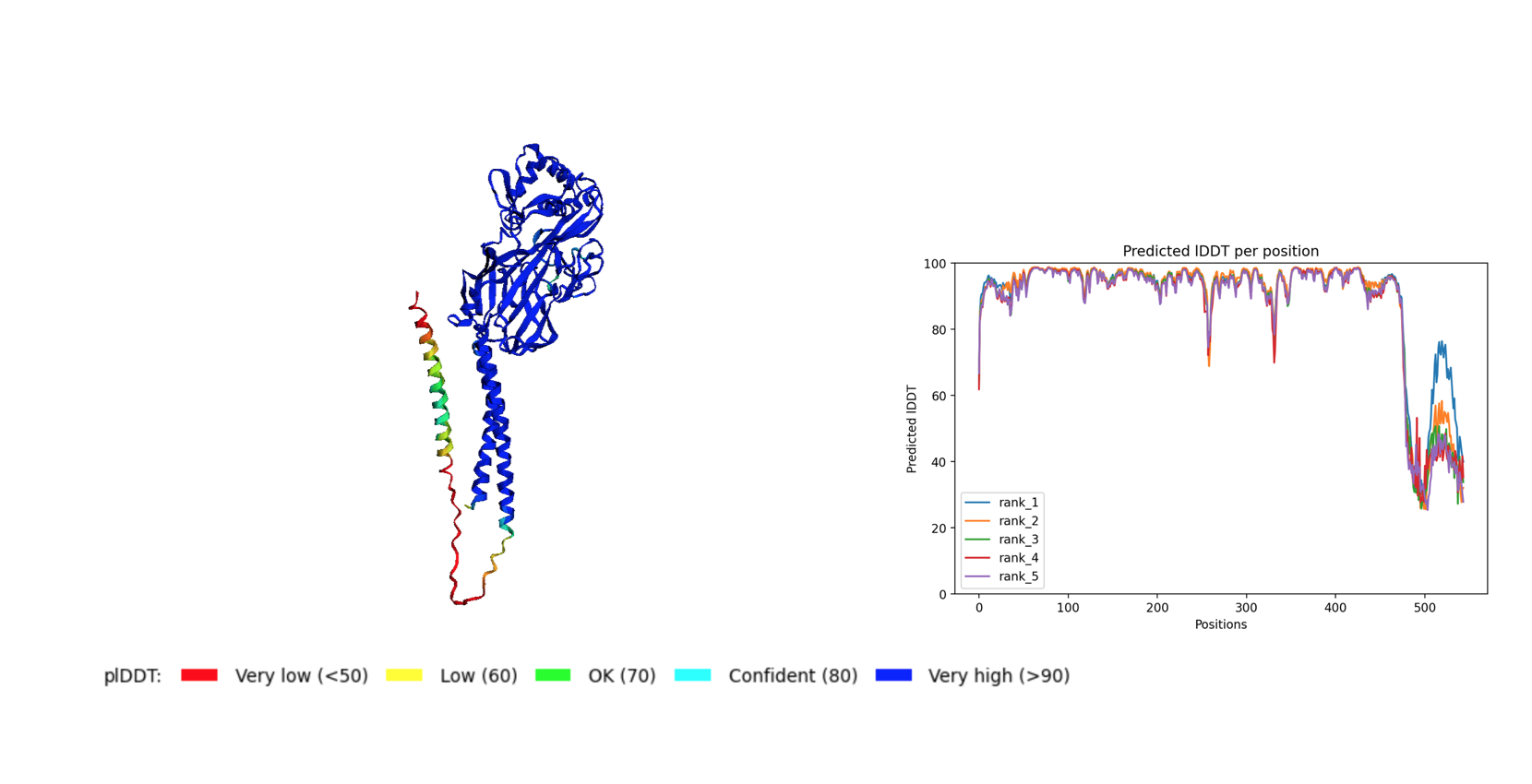
**
